# Comparison of Health Behaviors of Healthcare Workers and the General Public in Israel: A Cross-Sectional Survey

**DOI:** 10.3390/ijerph21030268

**Published:** 2024-02-27

**Authors:** Shira Ramot, Orna Tal, Tova Rosenbloom

**Affiliations:** 1 Department of Management, Health Systems Management Program, Bar Ilan University, Ramat Gan 5290002, Israel; ornatal@shamir.gov.il (O.T.); rosenbt1@biu.ac.il (T.R.); 2 Shamir Medical Center (Formerly Assaf Harofeh), Be’er Ya’akov 7033001, Israel; 3 ICET—Israeli Center for Emerging Technologies, Zerifin 7033001, Israel

**Keywords:** healthcare workers, health behaviors, physicians, nurses, health education

## Abstract

Healthcare workers (HCWs) are role models and advisors for promoting health behaviors among their patients. We conducted a cross-sectional survey to identify and compare the health behaviors of 105 HCWs and 82 members of the Israeli public. Of 13 health behaviors examined, undergoing screening tests, getting influenza vaccines and smoking were significantly different between the HCWs and the public. Further comparison between physicians and other HCWs (e.g., nurses, physiotherapists, dieticians) showed that the physicians reported the least favorable health behaviors: having less than 7 h of sleep, being less likely to eat breakfast, having greater alcohol consumption and being least likely to undergo regular screening tests. Analysis of a composite healthy lifestyle score (which included 11 health behaviors) showed statistically significant differences among the three groups (*p* = 0.034): only 10.6% of the physicians had a high healthy lifestyle score compared to the other HCWs (34.5%). In conclusion, the HCWs and the public report suboptimal health behaviors. Beyond the concern for HCWs’ personal health, their health behaviors have implications for the health of patients and the general public, as they play an important role in health promotion and counseling. HCWs’ suboptimal “health profile” mandates implementing policies to improve their knowledge of recommended health behaviors, primarily targeting physicians, even at an early phase of their professional journey.

## 1. Introduction

Modifiable health behaviors, such as tobacco use, physical inactivity, an unhealthy diet and alcohol consumption, all increase the risk of noncommunicable diseases such as cancer, coronary heart disease and diabetes. Although the health behavior of healthcare workers (HCWs) primarily affects their own well-being, HCWs also have a major effect as role models and advisors for promoting health behaviors among their patients [1]. The COVID-19 pandemic has emphasized the important status of HCWs as role models and agents of change. They are the most trusted advisors and influencers of vaccination decisions [2]. Medical sources were found to be the most influential source of information for decision making about vaccinations [3,4].

Studies have shown positive relationships between physicians’ and nurses’ own personal health behaviors, such as proper nutrition, screening tests and vaccination, and the provision of healthy lifestyle advice to their patients [5,6,7,8]. Physicians who believed that they were obligated to provide healthy lifestyle counselling to patients and had sufficient knowledge about healthy behaviors were more likely to provide patients with such advice [9].

Despite their professional experience, health knowledge and awareness, HCWs themselves do not always comply with health behavior recommendations [10,11,12,13]. Lack of physical activity and insufficient sleeping were among the suboptimal health behaviors reported by HCWs, including physicians, nurses, physician assistants and interns [8,14]. Additionally, physicians do not always adhere to screening tests recommendations even if they are aware to their importance [15].

Understanding the health behavior of HCWs compared to that of the public is essential for promoting the health of HCWs as well as their patients. The consequences of an unhealthy behavior among HCWs, like sleep deprivation, can adversely affect their performance, medical errors and, ultimately, patient care [8]. Such a comparison can also provide a broad view of the special status of HCWs: on the one hand they represent the healthcare system but on the other hand they may have their own views and perceptions as members of the public. In studies examining HCWs’ intention to get vaccinated, it was found that although HCWs are part of the health system, some of the barriers to getting vaccinated were actually similar to those of the general public [16]. Similarly, some of the barriers reported by physicians for not performing sufficient physical activity (PA), were also partly similar to those reported by the general population: lack of time, lack of motivation and lack of facilities [5].

The aim of this study was to identify and compare the “health profile” of HCWs and the public in Israel, including health-promoting behaviors (PA, healthy eating, sleep hygiene), health risk behaviors (smoking, excessive alcohol consumption) and health screening and vaccination behaviors.

## 2. Materials and Methods

### 2.1. Setting and Participants

A cross-sectional survey was conducted among a sample of HCWs and members of the public in Israel. Two participant populations were included in the study: (1) HCWs working at a general governmental 900-bed hospital in central Israel as well as HCWs in the final stage of a health manager training program and (2) members of the public. The questionnaires were completed anonymously. The study was approved by Shamir Medical Center’s ethics committee of (approval number ASF-0196-22) and conducted according to the guidelines of the Declaration of Helsinki.

### 2.2. Sample

The calculation of the sample size was conducted using G*Power version 3.1.9.7 software and based on the distributions of several variables. In general, we calculated the minimum sample size required (z test for differences between independent proportions). For the minimum proportions gap 0.2 (α = 0.05 and 1-β = 0.8), the minimum sample size needed for the study was 157 (101 HCWs and 56 members of the general public). Thus, the sample size of 187 participants was considered sufficient.

### 2.3. Study Tool

The self-administered electronic questionnaire was largely based on a questionnaire disseminated among Israeli physicians in a previous study [11]. After the initial construction of the questionnaire, it was completed by 15 respondents (10 HCWs and 5 members of the public) and revised according to their feedback. The final questionnaire comprised 3 sections (Supplementary File S1).

The first section evaluated the respondents’ health behaviors and included 8 items: (1) PA frequency—measured on a scale ranging from 1 (>5 times/week) to 4 (not at all) and PA duration/activity (in minutes); (2) nutritional habits—measured using five questions: the frequency of eating breakfast, the frequency of eating lunch, eating according to the principles of the Mediterranean diet, eating processed food and drinking sugar-sweetened beverages; the answers were provided on a scale ranging from 1 (every day) to 4 (less than once a week); (3) smoking (yes/no); (4) alcohol consumption frequency—measured on a scale between 1 (>2 times/week) and 5 (never); (5) the number of nightly hours of sleep; (6) perceived health status—measured on a scale ranging from 1 (excellent) to 5 (poor); (7) undergoing screening tests regularly—measured on a scale between 1 (to a large extent) and 4 (not at all); and (8) getting seasonal influenza vaccinations regularly (yes/no), getting the initial COVID-19 vaccinations (2 doses/third booster dose/fourth booster dose) and getting a hypothetical seasonal COVID-19 vaccination (yes/no).

The second section of the questionnaire was directed to HCWs only and included 2 questions on their perception of their professional role: (1) to what extent would you provide your patient with recommendations on healthy lifestyle habits (nutrition, PA)?; and (2) to what extent does your professional role (the fact that you are part of the health system) affect your decision to get vaccinated. The answers to both questions were provided on a scale ranging from 1 (to a large extent) to 4 (not at all).

The third section of the questionnaire included sociodemographic questions (age, gender, marital status, number of children, education, nationality, religion and income).

### 2.4. Data Collection

The questionnaire was disseminated online during the end of 2022 and the beginning of 2023 by email and WhatsApp. The sample of HCWs working at the medical center was obtained by sending emails to the medical center workers and the sample of HCWs from the health manager training program was obtained by sending a request to participate in the survey through the course’s Whatsapp group. The sample of members of the public was obtained using the snowball method using the researchers’ personal contacts.

### 2.5. Statistical Analysis

Statistical analysis was performed using SPSS version 29 (IBM Corporation, Armonk, NY, USA). The respondents were divided into two groups, HCWs and the general public, and, in the next step, into three groups: physicians and interns; other health professionals (HCPs) (like nurses, physiotherapists, dieticians); and the general public. The study populations’ characteristics and variables were summarized using descriptive statistics. Categorical variables were summarized by number and percentage and compared using a chi-squared test, and continuous variables were summarized by mean and standard deviation and compared by an independent *t*-test.

### 2.6. Healthy Lifestyle Score

To determine if the respondent followed a healthy lifestyle, we created a “healthy lifestyle” score summarizing 11 health behaviors and their components in the first section of the questionnaire (PA, nutrition, sleep, smoking status, alcohol consumption, undergoing screening tests and getting vaccinated). Each behavior was coded “1” if the respondent followed health behavior recommendations and “0” if the recommendations were not followed. The “healthy lifestyle” score could range from 1 to 11 for each participant. We then divided the “healthy lifestyle” into 3 categories: low (score 1–3), average (score 4–6) and high (score ≥ 7).

Health behavior recommendations were defined by others as follows: performing at least 150 min of PA in an average week; eating breakfast/lunch every day/almost every day, eating processed food and drinking sugar-sweetened beverages less than once a week; not smoking; sleeping for at least 7 h at night; drinking one serving or less of alcohol per month; undergoing screening tests to a large extent and getting vaccinated for influenza) [11,14,17,18,19,20,21,22].

## 3. Results

### 3.1. Sociodemographic Characteristics of the Study Population

A total of 187 respondents completed the survey and were included in the analysis: 105 (56%) HCWs and 82 members of the public (44%). The HCW group included 47 (25%) physicians and interns and 58 (31%) other healthcare professionals.

The respondents’ mean age was 45.9 ± 14.5 years (range 18–94). Two-thirds of the respondents were women (66%) and most (80%) were married. Most participants (85.6%) had an academic education. About half (54%) reported an average income and 28% reported an income above average (Table 1).

### 3.2. Health Behaviors of the Study Populations

Of the 13 health behaviors examined, undergoing screening tests, influenza vaccination and smoking were statistically significantly different between the HCWs and the general public, while sleeping showed a trend for a statistically significant difference between the groups (Table 2).

In the second stage of the analysis, we examined whether the health behavior of physicians and interns is different from that of other HCPs and of the general public. Surprisingly, eight health behaviors were found to be statistically significantly different among the three subgroups: eating breakfast, sleeping hours, smoking, alcohol consumption, screening tests, getting vaccinated against influenza and against COVID-19, and getting a hypothetical seasonal COVID-19 vaccine (Table 3). The mean PA time reported was 38.1 ± 28.4 min per week with no significant difference between the three groups, Therefore, on average, none of the respondents met the leisure time PA recommendations of at least 150 min per week.

The percentage of respondents who reported eating breakfast every day/almost every day was similar among the public (46.3%) and other HCPs (48.3%), while only 21.7% of the physicians and interns reported eating breakfast every day/almost every day and 41.3% of them reported eating breakfast less than once a week (Table 3).

The number of recommended sleeping hours at night (≥7) was met by only 25.1% of the respondents. Most of the physicians and interns (76.6%) reported sleeping less than 7 h, compared to 56.9% of the other HCPs and 50.6% of the public.

About a third of the respondents (34.2%) reported undergoing screening tests to a small extent, with similar proportions among the public and other HCPs compared to half of the physicians and interns (51.1%). Most of the physicians and interns (80.9%) reported getting vaccinated for influenza, compared to 39.7% of the other HCPs and 36.6% of the public. More than half of the physicians were vaccinated with the fourth COVID-19 vaccine, compared to less than a fifth of the other HCPs and members of the public (17.2% and 18.3%, respectively). Interestingly, most of the physicians and interns (88.3%), half of the other HCPs (50.8%) and two-thirds of the public (66.6%) reported that they would get vaccinated with a seasonal COVID-19 vaccine. About a third of the physicians and interns (36.2%) reported drinking alcohol at least twice a week, compared to 13.7% of the other HCPs and 26.8% of the public. Only 4.3% of the physicians and interns and 5.2% of the other HCPs reported smoking compared to 17.3% of the public.

In general, the physicians and interns reported the least favorable health behaviors: getting less than 7 h of sleep, being less likely to routinely eat breakfast compared to the other HCPs and to the public, consuming more alcohol than the other HCPs and undergoing screening tests regularly the least.

### 3.3. Combined Healthy Lifestyle Score

A statistically significant difference in healthy lifestyle score categories was observed among the three groups (Table 4). Seventy percent of the physicians and interns, 50% of the other HCPs and 62.2% of the public had an average healthy lifestyle score. A third of the other HCPs (34.5%) were classified as having a high healthy lifestyle score while the proportion of physicians and interns with a high healthy lifestyle was the lowest among the three subgroups (12.8%).

### 3.4. Recommendation to Patients and the Influence of the Health System on the Decision to Get Vaccinated

Most HCWs (81%) reported that they would recommend, to a great extent, healthy lifestyle behaviors to their patients. Examining the “healthy lifestyle” score among this group revealed that 18% of them reported performing <3 health out of the 11 behaviors while 57% of them reported performing 4–6 of the health behaviors. Almost two-thirds (61%) of the HCWs reported that their decision to get vaccinated was affected to a great extent by their being part of the health system (65% of the physicians and interns and 62% of the other HCPs).

## 4. Discussion

This study presents a snapshot of the health behavior of HCWs in general and physicians and medical interns in particular. The results of this study demonstrate differences in health behaviors between HCWs and the general Israeli public. Specifically, undergoing screening tests, getting vaccinated for influenza and smoking were significantly different between these two groups. Moreover, both the public and HCWs did not report compliance with recommended health behaviors. Our findings show that physicians and interns do not always comply with the recommendations for a healthy lifestyle. In fact, among the three subgroups examined in our study, the physicians and interns reported the least favorable health behaviors: on average they did not meet the weekly PA recommendations, most of them reported sleeping less than 7 h at night, they were less likely to routinely eat breakfast compared to other HCPs and to the general public, they reported consuming more alcohol than the other HCPs and they do not tend to undergo regular screening tests regularly. These findings corroborate the findings of Wilf-Miron et al. [11], which demonstrated non-optimal health behavior among 4832 Israeli physicians: 21% reported poor health, 57% were overweight, 79% did not meet the nutritional recommendations and a quarter of them did not meet the recommendations for sleeping hours. Similar results were also found in studies of physicians in other countries. In a study conducted in Poland, only 11% of physicians met the recommendations of five factors for a healthy lifestyle that were examined (alcohol consumption, smoking, weight, nutrition and physical activity) [9]. A study conducted in Ireland showed that the health behavior of physicians was better than that of the general public, but the consumption of alcohol and physical activity required improvement [23]. Skipping breakfast, which was highly prevalent among the physicians in the study, is also consistent with other studies [24,25]. Lack of time was the most common reason reported by physicians among the barriers to eating a meal regularly [26]. Bazargan et al. have found that age and multiple working hours predict skipping breakfast [27]. Therefore, the nature and working conditions of physicians pose a challenge in maintaining a healthy diet that includes eating breakfast.

In accordance with the findings of the current study, which showed that most HCWs and physicians do not meet the recommendations for sleeping 7 h at night, other studies have demonstrated insufficient sleep among physicians [11,12,28], with an average of 5.9 to 6.5 h of sleep per day [28]. Insufficient sleep among physicians can be explained by the demanding nature of their work, which includes long working hours and shift work [29]. Most of the physicians in our survey reported undergoing screening tests only to a low extent or not at all. This is consistent with other studies’ findings [30]. In a study conducted among hospital-based physicians in Israel, less than half of physicians actually underwent the tests themselves, although 94% of them reported that they believe in the importance of screening tests for physicians [15]. Similar findings were reported in other countries regarding non-adherence to regular screening for colorectal, cervical and breast cancer [31,32,33]. One of the barriers mentioned by physicians was lack of time. It was also noted that physicians have a tendency to deny disease and medical conditions, and relate to their own health only when they are seriously ill [34].

Studies of lifestyle behaviors in nurses have also shown a pattern of non-adherence to public health guidelines such as PA, sedentary behavior, nutrition, smoking and alcohol consumption, although health promotion is an essential part of their work [35,36]. In a study conducted among nurses in Israel, most reported poor nutritional habits and two-thirds did not meet the recommendations for PA and reported sleeping less than 7 h at night [37]. In this study, nurses and other HCPs did not comply with the recommendations for weekly PA, most of them did not get vaccinated against influenza and only 17.2% got vaccinated with the fourth dose of the COVID-19 vaccine. The rate of smokers among nurses and other HCPs was higher than that of physicians. These findings are consistent with other studies that examined health habits among nurses and showed a pattern of non-compliance with guidelines for a healthy lifestyle including physical activity. High job stress was found to be main negative factor affecting health screening and health-promoting behaviors among nurses [35,37,38]. Our study demonstrated non-compliance with physical activity recommendations among the entire sample, including physicians, in accordance with other studies from around the world and in Israel [11,12,23].

Due to cultural and methodological differences, it is difficult to compare our findings regarding the alcohol consumption of HCWs and the public to those reported in other studies [39]. However, similarly to our findings, other studies have also reported high consumption of alcohol among physicians compared to the general public [28,40]. In studies conducted in five countries, high alcohol consumption was found to be associated with age, gender and multiple working hours [40]. Another explanation given for the high consumption among physicians was the use of alcohol as a coping mechanism [28].

Our findings also corroborate those of other studies that have also reported lower levels of smoking among physicians compared to nurses [37,41]. The awareness of smoking-related morbidity and fear related to the consequences of lung cancer may explain this. In Israel, as in other developed countries, health authorities’ recommendations for vaccination, including seasonal influenza vaccination, are met with only partial compliance by HCWs [42,43,44]. In this study, the physicians reported a higher rate of influenza and COVID-19 vaccinations compared to the other HCPs and the public. This finding is in agreement with data from the Israeli Ministry of Health on the influenza vaccination rate of medical staff, which also showed that the vaccination rate among nurses is lower than that of physicians. In the same study, physicians perceived the influenza vaccine as less harmful compared to other HCPs [44]. Our study showed that HCWs also showed hesitancy toward COVID-19 vaccines. The intention to get vaccinated against COVID-19 varied with gender and profession [45]. Nurses were four times more likely to delay COVID-19 vaccination compared to physicians [46]. A study conducted during lockdown in Israel before the COVID-19 vaccine was distributed had reported that the level of willingness to be vaccinated was higher among physicians than nurses [47]. Another study conducted in Israel has reported that 53.9% of physicians were unwilling to get vaccinated with the fourth COVID-19 booster dose, compared to 83.3% of nurses [48].

Our findings regarding suboptimal compliance with the recommendations for PA, a low rate of influenza vaccination and a low rate of undergoing screening tests among the public are consistent with Israel Ministry of Health data. According to a national health survey conducted by the National Center for Disease Control between 2018 and 2020, two-thirds of participants did not meet the recommendations for PA and only 40% of those eligible underwent a colon cancer test [49]. In addition, according to the Ministry of Health’s report for the 2019/2020 influenza season, only 25.3% of the population in Israel were vaccinated against influenza (67.9% over the age of 65) [50].

The reasons related to the nature and working conditions of physicians can explain some of the unfavorable health behaviors of HCWs, especially physicians. In a survey preformed among hospital staff in the United Kingdom, HCWs lacked the motivation for healthy behaviors and cited their overall fatigue and lack of facilities at work as reasons for poor PA [51]. Beyond that, it was also suggested that sometimes physicians feel protected due to their medical knowledge [52]. In addition, the COVID-19 pandemic, which was a great challenge for HCWs, with increased workload and stress, had also led to changes in their health behaviors [53].

As to counseling patients on healthy habits, it is known that HCWs perceive themselves as role models and health advisors [54]. At the same time, the relationship between physicians’ personal health lifestyle practices and the rates of patient counseling may not be straightforward [55]. In our study, although most of the HCWs reported that they advise patients on healthy lifestyles to a great extent, the health behavior of some of them was suboptimal. Counseling healthy lifestyles regardless of HCWs’ own health habits was also reported in other studies. In a study that dealt with obesity, it was found that sometimes the physicians were not aware of their own obesity [56]. It is possible that some of the HCWs in our study were not aware of their suboptimal health behavior and perceived themselves as maintaining a healthy lifestyle and therefore felt that they can also advise their patients. Despite its importance to general health and well-being, nutrition is not part of the curriculum of medicine and nursing programs in Israel [57]. Furthermore, as the HCW sample in this study work at a hospital, they may not have time to provide lifestyle advice due to the short encounter with patients that is characteristic of acute medical situations in hospital settings.

Another explanation that can be offered is related to the special status of HCWs: on the one hand they are part of the healthcare system and hence feel they have a responsibility as role models, but on the other hand they are individuals who are part of a general public who sometimes does not comply with the recommendations for a healthy lifestyle due to lack of time or motivation. Experiencing what it means to struggle with poor health behavior can make physicians more empathetic to their patients who struggle with the same issues, thus increasing counseling rates [55].

### 4.1. Policy Implications

To fundamentally address the problem of HCWs’ suboptimal health habits, it is necessary to raise their awareness and knowledge of recommended health behaviors and to promote their health and well-being on several levels. This should start early in their professional education during medical school to implement favorable habits as well as to enhance their role as agents of change as therapists. At the national level, HCWs’ health should be defined as a priority as part of a national plan with incentives for health-promoting medical institutions and organizations. In the United Kingdom (UK), there are various national initiatives to improve the health and well-being of National Health Services (NHS) staff. For example, the NHS Healthy Workforce Program, established in 2016, focused on implementing employer-led health and well-being initiatives as well as creating health supportive organizational practices and culture. Organizations that had introduced workplace health and well-being initiatives, such as PA, healthier food choices, support for mental health and increasing influenza vaccination uptake by staff, received payments [58].“Healthy Israel 2020”, an Israeli Ministry of Health initiative setting health behavior goals to be achieved by 2020, recommended the integration of lifestyle interventions into the academic curricula for health professionals including medical students, in addition to residency programs and continuing medical education [59].

At the organizational level, the workplace is a preferred setting for promoting health among HCWs. Although the importance of workplace health promotion interventions is well recognized [60], it can be challenging due to HCWs’ working conditions, high workload, heterogeneity of work activities and long work hours [61]. Interventions among HCPs to improve health behaviors such as physical activity, diet, smoking status, mental health and stress were effective to various degrees [62]. Lifestyle health promotion interventions for nurses that targeted diet, body composition, PA or stress provided positive outcomes for nurses’ health and/or well-being [35]. Interventions that comprised only educational materials were the least effective in changing HCWs’ behavior, while those that took a systematic approach were the most effective [8]. Such a systematic approach includes identifying staff needs at all levels, engaging upper-level staff members and management and providing flexible programs that enable the participation of a diverse staff group [8]. Several strategies and characteristics that produced successful health outcomes among HCWs were reported in the literature and are therefore recommended as follows.

(1) Whole-system approaches are recommended in line with a review commissioned by the UK Department of Health to address the health and well-being at work of HCWs [63], which highlighted the need for whole-system interventions. It proposed five system-level changes for healthcare workplaces: understanding local staff needs, staff engagement at all levels, strong visible leadership, support for health and well-being at senior management and board level, and a focus on management capability and capacity to improve staff health and well-being. Accordingly, interventions should include efforts to involve the management regarding activities to promote health, such as discussion groups and a steering committee, as well as the involvement of HCWs themselves in the planning and implementation of the interventions. In an intervention program to promote the health of HCWs at a hospital in Israel, both the management and the HCWs were involved [64].
(2) Best evidence is available for multi-component interventions combining behavioral/educational interventions (e.g., counseling, online or face-to-face lectures, educational material), environmental interventions (e.g., walking routes, availability of healthy food items at work) and organizational support (e.g., reduced work hours to promote activity or good sleeping, offering interventions like exercise classes within work hours or incentives) [65,66]. According to a systematic review, multi-component strategies, motivational strategies and financial incentives were effective in improving HCWs’ health in the workplace [67]. A multidisciplinary work health promotion program that included exercise classes, one-on-one nutrition consultation and behavioral education showed improved anthropometric and physical fitness profiles among HCWs [61].
(3) Motivational strategies were effective in motivating change, including the use of staff members who considered themselves a role model to their peers and whose aim was to influence health practices. These influencers also tended to increase their own positive health behaviors [68].
(4) Positive health behaviors can be increased through financial incentives, such as subsidies for healthy food or gyms. In a survey conducted at Massachusetts General Hospital, it was shown that social norms plus small financial incentives increased HCWs’ healthy food choices [69].
(5) The intensity of activities within the intervention plan is important. In a narrative review of obesity prevention interventions for HCWs, it was found that moderate-to-intense behavioral strategies (such as daily or weekly counseling/training sessions delivered by a trained professional) appeared to improve weight outcomes better than less intensive strategies [66]. A higher intensity of contact has been also shown to increase the effectiveness of PA interventions. Optimizing the intensity of contact with participants can be carried out by using technologies [62] such as smart phone apps.
(6) Widening activities beyond the workplace to include family and friends may further enhance engagement and improvements of healthy behavior [70].
(7) Increasing knowledge and awareness of healthy behavior among HCWs is recommended for their own health and to encourage them to be role models for healthy lifestyles for their patients. Universities and professional societies should include lifestyle education in the training of all healthcare professionals. This is one of the US national physical activity plan strategies [71]. Lifestyle improvement under the guidance of medical professionals has been found to be effective in promoting health [59]. However, although Israeli family medicine residents consider counseling patients about a healthy lifestyle to be an integral part of their work, they do not feel well prepared to do so [72], emphasizing the need for the integration of training on healthy lifestyle into the curriculum of medical schools [59,72].

Physicians’ representative organizations have an important role in the effort to improve HCWs’ health. The Israeli Society of Lifestyle Medicine (ISLM) was established in 2012 under the Israel Association for Family Physicians and is an active member of the Israeli Medical Association (IMA). As part of its activities, the ISLM promotes educational activities to promote physicians’ health and well-being initiatives [59]. They have created a syllabus and an online course on lifestyle medicine for physicians and medical students. The IMA established a physicians’ health and resilience forum in 2015, which provides information (articles, podcasts) on its website, free or subsidized activities and workshops in various lifestyle areas such as PA, nutrition, healthy sleep, smoking cessation and mental resilience [73].

Finally, to improve compliance with getting influenza vaccines, undergoing screening tests and following PA recommendations among the general public, efforts should be made to develop effective health promotion interventions. Regarding cancer screening, interventions can increase awareness and knowledge about cancer prevention and reduce and overcome barriers to their uptake [74]. As individuals’ decision to receive influenza vaccination is influenced by several factors, such as perceptions of vaccine efficacy, vaccine safety and adverse events, as well as advice from health professionals [75], HCWs have an important role in raising awareness and knowledge among the public that will lead to a greater rate of vaccination.

### 4.2. Limitations

The study has some limitations. First, convenience samples of HCWs and the general public were used; therefore, the study results may not be generalizable to the entire Israeli population or to all HCWs. Yet even this sample provides significant information about HCWs’ health behaviors. A study with a representative sample of the population and a sample of HCWs from the entire health system may present a more generalized picture. The small sample and low response rate of HCWs could be explained by the high workload in hospitals and burnout of staff. Other studies have also reported low response rates among HCWs. The percentage of women among the respondents was relatively high; however, this represents the population of HCWs around the world [76]. Online surveys have many advantages, as they have the potential to recruit large numbers of respondents at a low cost. However, online surveys may be associated with selection bias, since people who do not have access to the Internet or those who do not have digital literacy have a zero probability of selection. We can assume that HCWs have digital literacy; therefore, they may not have been affected by this type of selection bias, but it may have affected the general public. Finally, the questionnaire was based on self-reporting. Although anonymity was assured, some responses may have been subject to social desirability bias. It is possible that in relation to health behaviors in future research, objective measures such as body mass index can also be incorporated.

## 5. Conclusions

This study showed a snapshot of the health behavior of HCWs and the general Israeli public. The results of the study suggest that health behavior differs between HCWs and the general public, with both groups reporting suboptimal health behavior. Beyond the concern for the personal health of HCWs, the study’s findings have implications for the health of patients and the public as a whole, as HCWs play an important role in health promotion and health counseling. Identifying health behaviors that are not in accordance with the recommendations can help in the implementation of intervention plans and strategies to improve health habits.

The indication for the suboptimal “health profile” of HCWs that emerged in the study mandates implementing policies to improve HCWs’ knowledge of recommended health behavior. Policies that can help to achieve this goal include providing specific education and training, starting from medical school and nurse training programs, creating a culture of health in the workplace, creating opportunities for physical activity and regular meals, and encouraging vaccination and regular screening tests. Consequently, this will make an additional contribution to improving the health behaviors of the public.

## Figures and Tables

**Table 1:** Respondents’ sociodemographic characteristics.

Variable	Physicians and Interns	Other Healthcare Professionals	Members of the Public	All
	N = 47	N = 58	N = 82	N = 187
Age, years, mean (SD)	43.5 (13.9)	43 (10.7)	49 (16.4)	45.9 ± 14.5
Sex, n (%)				
Male	24 (51.0%)	2 (3.4%)	37(45.1%)	63 (33.6%)
Female	23 (48.9%)	56 (96.5%)	45 (54.8%)	124 (66.3%)
Marital status, n (%)				
Married/living with a partner	40 (85.1%)	46 (79.3%)	65 (79.3%)	151 (80.7%)
Single/divorced/widowed	7 (14.8%)	12 (20.6%)	17 (20.7%)	36 (19.3%)
Number of children, mean (SD)	2 ± 1.7	3 ± 1.7	3.3 ± 2.1	2.02 ± 1.7
Religion, n (%)				
Jewish	44 (93.6%)	57 (98.3%)	81 (98.7%)	182 (97.3%)
Muslim	3 (6.4%)	1 (1.7)	1 (1.2%)	5 (2.7%)
Religiosity, n (%)				
Secular	35 (74.4%)	22 (37.9%) *	30 (36.6%)	87 (46.5%)
Traditional	5 (10.6%)	10 (17.2%)	12 (14.6%)	27(14.4%)
Religious	6 (12.8%)	24 (41.4%)	39 (47.6%)	69 (36.9%)
Orthodox Jewish	1 (2.1%)	1 (1.7%)	1(1.2%)	3 (1.6%)
Education, n (%)				
Elementary school/high school	0	0	15 (18.3%)	15 (8.0%)
Higher education (non-academic)	0	2 (3.4%)	10 (12.2%)	12 (6.4%)
Bachelor’s degree	0	32 (55.1%)	31 (37.8%)	63 (33.6%)
Master’s degree or higher	47(100%)	24 (41.4%)	26 (31.7%)	97 (51.8%)
Income, n (%)				
Below average	11 (23.4%)	5 (8.6%)	16 (19.5%)	32 (17.1%)
Average	24 (51.0%)	37 (63.8)	41 (50%)	102 (54.5%)
Above average	12 (25.5%)	16 (27.6%)	24 (29.2%) *	52 (27.8%) *
Perceived health status, mean ± SD	2 ± 0.7	2.1 ± 0.7	2.2 ± 0.8	2.1 ± 0.7
Excellent	11 (23.4%)	9 (15.1%)	16 (19.5%)	36 (19.3%)
Very good	27 (57.4%)	34 (58.6%)	37 (45.1%)	98 (52.4%)
Good	7 (14.9%)	13 (22.4%)	23 (28%)	43 (23%)
Poor	2 (4.3%)	2 (3.4%)	6 (7.3%)	10 (5.3%)

SD = standard deviation, * missing = 1.

**Table 2:** Comparison of health behaviors of HCWs and the general public. Only statistically significant differences are shown.

Health Behavior	Levels	HCWs N = 105	Members of the Public N = 82	All N = 187	*p*-Value
Smoking	Yes	5 (4.8%)	14 (17.3%)	19 (10.1%)	0.005
Hours of sleep	>8	3 (2.9%)	8 (9.9%)	11 (5.9%)	0.05
	7 h	33 (31.4%)	32 (39.5%)	65 (34.9%)	
	6 h	45 (42.9%)	31 (38.3%)	76 (40.6%)	
	≤5 h	24 (22.9%)	10 (12.3%) *	34 (18.8%) *	
Screening tests	To a large extent	26 (24.8%)	13 (15.9%)	39 (20.8%)	0.013
	To an average extent	16 (15.2%)	27 (32.9%)	43 (23%)	
	To small extent	42 (40%)	22 (26.8%)	64 (34.2%)	
	Not at all	21 (20%)	20 (24.4%)	41 (21.9%)	
Influenza vaccination	Yes	61 (58.1%)	30 (36.6%)	91 (48.7%)	0.003
	No	44 (41.9%)	52 (63.4%)	96 (51.3%)	

* missing = 1.

**Table 3:** Comparison of health behaviors among physicians and interns, other health professionals and the general public.

Health Behavior	Levels	Physicians and Interns N = 47	Other Health Professionals N = 58	Members of the Public N = 82	All N = 187	*p*-Value
Breakfast	Every day/almost every day	10 (21.7%)	28 (48.3%)	38 (46.3%)	76 (40.6%)	0.026
	3–4 times/week	8 (17.4%)	7 (12.1%)	6 (7.3%)	21 (11.2%)	
	1–2 times/week	9 (19.6%)	12 (20.7%)	20 (24.4%)	41 (21.9%)	
	>1 time/week	19 (41.3%) *	11 (19%)	18 (22%)	48 (25.6%) *	
Alcohol Consumption	>2 times/week	7 (14.9%)	2 (3.4%)	11 (13.4%)	20 (10.6%)	0.018
	2 times/week	10 (21.3%)	6 (10.3%)	11 (13.4%)	27 (14.4%)	
	2–4 times/month	10 (21.3%)	4 (6.9%)	15 (18.3%)	29 (15.5%)	
	≤1 time/month	11 (23.4%)	27 (46.6%)	21 (25.6%)	59 (31.5%)	
	Never	9 (17.3%)	19 (32.8%)	24 (29.3%)	52 (27.8%)	
Smoking	Yes	2 (4.3%)	3 (5.2%)	14 (17.3%)	19 (10.1%)	0.02
Hours of sleep	≥7 h	11 (23.4%)	25 (43.1%)	40 (49.4%)	76 (40.8%)	0.018
	6 h	21 (44.7%)	24 (41.4%)	31 (38.3%)	76 (40.6%)	
	≤5 h	15 (31.9%)	9 (15.5%)	10 (12.3%) *	34 (18.8%) *	
Screening tests	To a large extent	8 (17%)	18 (31%)	13 (15.9%)	39 (20.8%)	0.005
	To an average extent	4 (8.5%)	12 (20.7%)	27 (32.9%)	43 (23%)	
	To small extent	24 (51.1%)	18 (31%)	22 (26.8%)	64 (34.2%)	
	Not at all	11 (23.4%)	10 (17.2%)	20 (24.4%)	41 (21.9%)	
Influenza vaccination	Yes	38 (80.9%)	23 (39.7%)	30 (36.6%)	91 (48.6%)	<0.001
COVID-19 vaccination	Fourth dose	25 (53.2%)	10 (17.2%)	15 (18.3%)	50 (26.7%)	<0.001
	1 + 2+3 doses	19 (40.4%)	36 (62.1%)	55 (67.1%)	110 (58.8%)	
	1 + 2 doses	3 (6.4%)	11 (19%)	10 (12.2%)	24 (12.8%)	
	No	0	1 (1.7%)	2 (2.4%0	3 (1.6%)	
Seasonal COVID-19 vaccine	Yes	38 (88.3%)	29 (50.8%)	54 (66.6%)	121 (67%)	<0.001
PA frequency, n (%)	>5 time/week	2 (4.3%)	3 (5.3%)	6 (7.3%)	11 (5.9%)	0.8
	3–5 times/week	14 (29.8%)	16 (28.1%)	25 (30.5%)	55 (29.6%)	
	1–2 times/week	23 (48. 9%)	22 (38.6%)	35 (42.7%)	80 (43%)	
	Never	8 (17%)	16 (28.1%) *	16 (19.5%)	40 (21.5%) *	
PA duration, minutes, mean ± SD		38.62 ± 28.4	35.26 ± 27.9	39.88 ± 28.44	38.13 ± 28.41	0.6
Lunch	Every day/almost every day	20 (42.6%)	33 (56.9%)	51 (62.2%)	104 (55.6%)	0.3
	3–4 times/week	14 (29.8%)	9 (15.5%)	15 (18.3%)	38 (20.3%)	
	1–2 times/week	10 (21.3%)	12 (20.7%)	12 (14.6%)	34 (18.2%)	
	>1 time/week	3 (6.4%)	4 (6.9%)	4 (4.9%)	11 (5.9%)	
Mediterranean diet	Every day/almost every day	21 (44.7%)	30 (51.7%)	25 (30.5%)	76 (40.6%)	0.2
	3–4 times/week	16 (34%)	14 (24.1%)	29 (35.4%)	59 (31.6%)	
	1–2 times/week	6 (12.8%)	8 (13.8%)	18 (22%)	32 (17.1%)	
	>1 time/week	4 (8.5%)	6 (10.3%)	10 (12.2%)	20 (10.7%)	
Eating processed food	Every day/almost every day	3 (6.4%)	5 (8.6%)	5 (6.2%)	13 (7%)	0.1
	3–4 times/week	10 (21.3%)	7 (12.1%)	12 (14.8%)	29 (15.6%)	
	1–2 times/week	10 (21.3%)	27 (46.6%)	32 (39.9%)	69 (37.1%)	
	>1 time/week	24 (51.1%)	19 (32.8%)	32 (39.9%) *	75 (40.3%)	
Drinking sweet drinks	Every day/almost every day	2 (4.3%)	3 (5.2%)	5 (6.1%)	10 (5.3%)	
	3–4 times/week	1 (2.1%)	3 (5.2%)	5 (6.1%)	9 (4.8%)	0.9
	1–2 times/week	8 (17%)	11 (19%)	18 (22%)	37 (19.8%)	
	>1 time/week	36 (76.6%)	41 (70.7%)	54 (65.9%)	131 (70%)	

* missing = 1.

**Table 4:** Healthy lifestyle score among study participants.

Healthy Lifestyle Score Categories	Physicians and Interns	Other Health Professionals	Members of the Public	All	*p*-Value
	N = 47	N = 58	N = 82	N = 187	
Low (≤3 health behaviors)	9 (19.1%)	9 (15.5%)	17 (20.7%)	35 (18.7%)	0.034
Average (4–6 health behaviors)	33 (70.2%)	29 (50%)	51 (62.2%)	113 (60.4%)	
High (≥7 health behaviors)	5 (10.6%)	20 (34.5%)	14 (17.1%)	39 (20.9%)	

## Data Availability

The raw data supporting the conclusions of this article will be made available by the authors on request.

## Supplementary Materials

The following supporting information can be downloaded at: https://www.mdpi.com/article/10.3390/ijerph21030268/s1, questionnaire.
