# Supplementary material for: Comparison of Health Behaviors of Healthcare Workers and the General Public in Israel: A Cross-Sectional Survey
Source: Int J Environ Res Public Health. 2024 Feb 27;21(3):268. doi: 10.3390/ijerph21030268 (PMC10970418; doi:10.3390/ijerph21030268)
Supplement: Supplementary file 1 [file ijerph-21-00268-s001.zip › ijerph-2813454-supplementary.pdf]

Supplementary File S1: English translation of the study questionnaire

A survey on health behaviors of healthcare workers and the public

Part 1

1. **In a typical week, how many times do you perform physical activity such as walking, swimming, running, cycling, body-building exercises, ball games, dance or yoga?**

|1| Not at all

|2| 1-2

|3| 3-5

|4| more than 5 times.

2. **On average, how long is each episode of activity? ______ minutes**

Which of the following questions (numbers 3-7) best describes your nutrition and eating habits?

3. **Eating breakfast**

|1| Daily or almost daily

|2| 3-4 times a week

|3| 1-2 times a week

|4| Less than once a week

4. **Eating lunch during the working day**

|1| Daily or almost daily

|2| 3-4 times a week

|3| 1-2 times a week

|4| Less than once a week

5. **Adherence to the principles of a Mediterranean diet: intake of vegetables, fruit, legumes, olive oil, nuts, unrefined wheat, fish, poultry and low-fat dairy products**

|1 Daily or almost daily

|2| 3-4 times a week

|3| 1-2 times a week

|4| Less than once a week

6. **Eating processed food products**

|1 Daily or almost daily

|2| 3-4 times a week

|3| 1-2 times a week

|4| Less than once a week

7. **Drinking sugar-sweetened beverages, including fruit juices**

|1|Daily or almost daily

|2| 3-4 times a week

|3| 1-2 times a week

|4| Less than once a week

8. **At present, do you smoke cigarettes?**

|1| No

|2| Yes

9. How often do you drink alcoholic beverages? (not including drinking wine for religious purpose "kidush")

|1 Not at all

|2| Once a month or less

|3| 2-4 times a month

|4| Twice a week

|5| More than twice a week

10. In the last month, how many hours, on average, did you spend sleeping at night?

|1| 5 or less

|2| 6

|3| 7

|4| 8 or more

11. To what extent do you regularly perform screening tests ? (fecal occult blood test, mammography)

|1| Do not perform at all.

|2| small extent

|3| Moderate extent

|4| Large extent

12. Are you regularly vaccinating against influenza?

|1| No

|2| Yes

13. Did you get Covid-19 vaccine?

|1| No

|2| Yes, 2 doses.

|3| Yes, including third booster

|4| Yes, including fourth booster

14. In general, how would you define your health status?

|1| Excellent

|2| Very good

|3| Good

|4| Fair

|5| Poor

Part 2

For healthcare workers:

15. What is your profession?

|1 Physician

|2| Intern

|3| Nurse

|4| Health profession

|5| Other

16. To what extent would you provide your patient with recommendations on healthy lifestyle habits (nutrition, PA)?

|1| To a large extent

|2| Moderate extent

|3| Small extent

|4| Not at all

17. **To what extent your professional role (the fact that you are part of the health system) affects your decision to get vaccinated**?

|1| To a large extent

|2| Moderate extent

|3| Small extent

|4| Not at all

Part 3

18. **Year of birth__________**

19. Sex

|1| Male

|2| Female

20. Marital status

|1| Married/ living with a partner

|2| Divorced

|3| Single

|4| Widowed

21. Number of children__________

22. Education

|1 Elementary school/ high school

|2| Higher education (non- academic)

|3| Bachelor's degree

|4| Master's degree or higher

23. Religion

|1|Jewish

|2| Muslim

24. Religiosity

|1| Secular

|2| Traditional

|3| Religious

|4| Orthodox-Jewish

25. Income

|1| Below average

|2| Average

|3| Above average
